# *In Vitro* Bactericidal Effects of Photodynamic Therapy Combined with Four Tetracyclines against *Clostridioides difficile* KCTC5009 in Planktonic Cultures

**DOI:** 10.3390/pathogens9040279

**Published:** 2020-04-11

**Authors:** Sung Sook Choi, Hui Yeong Oh, Eui Jin Kim, Hae Kyung Lee, Hyung Keun Kim, Hyun Ho Choi, Sang Woo Kim, Hiun Suk Chae

**Affiliations:** 1 College of Pharmacy, Sahmyook University, Seoul 01795, Korea; sschoi@syu.ac.kr; 2 Internal Medicine Uijongbu St. Mary’s Hospital, College of Medicine, The Catholic University of Korea, Seoul 06591, Korea; ohy880517@naver.com (H.Y.O.); Jin2kim6999@daum.net (E.J.K.); hykim@catholic.ac.kr (H.K.K.); chlgg@catholic.ac.kr (H.H.C.); viper@catholic.ac.kr (S.W.K.); 3 Department of Laboratory Medicine Uijongbu St. Mary’s Hospital, College of Medicine, The Catholic University of Korea, Seoul 06591, Korea; hkl@catholic.ac.kr

**Keywords:** *Clostridioides difficile* infection (CDI), photodynamic therapy (PDT), UVA, tetracyclines, chitosan, disinfection, disinfectants

## Abstract

Surface disinfection in health-care facilities is critical to prevent dissemination of *Clostridioides difficile* (*C. difficile*). Tetracyclines (TCs) are broad-spectrum antibiotics that are associated with a low risk of development of *C. difficile* infection (CDI) and are used as photosensitizers (PS) in photodynamic therapy (PDT). We evaluated whether TCs may be useful environmental cleansing agents. We compared the in vitro ability to kill *C. difficile* of four TCs (TC, doxycycline, minocycline, and tigecycline) combined with PDT using ultraviolet A (UVA). We included chitosan, a cationic material, as a booster to increase the photodynamic bactericidal efficacy of TCs. PDT-induced bactericidal effects were assessed by the number of viable cells and the degree of DNA damage and membrane integrity. To avoid the intrinsic antibacterial activity of TCs at high concentrations, we used low concentrations of TCs (0.05 and 0.1 mg/mL). The bactericidal effect of treatment with chitosan plus PDT was over 100 times higher than that with PDT alone for each of the four TCs. DNA damage measured by ethidium bromide monoazide and real-time quantitative polymerase chain reaction was also greater for PDT plus chitosan treatment than for PDT alone or under control conditions: the threshold cycle (Ct) values for the control, PDT, and PDT plus chitosan were 14.67 ± 0.22, 20.46 ± 0.12, and 25.54 ± 0.17, respectively. All four TCs caused similar levels of severe cell membrane damage during PDT compared with control conditions. These data suggest that PDT combined with any of the four TCs plus chitosan might be an available tool to kill efficiently planktonic form of *C. difficile*.

## 1. Introduction

*Clostridioides difficile* (*C. difficile*) is a spore-forming anaerobe that is the causative agent of severe and recurrent pseudomembranous colitis [1,2,3,4]. Toxins A and B are the major virulence factors of *C. difficile* infection (CDI) related to high morbidity and mortality [5]. Vancomycin and metronidazole are currently prescribed to treat CDI. Metronidazole, which is used for the treatment of mild to moderate infection, has an increasing failure rate. Oral vancomycin is the drug of choice for severe infection. It has few side effects, but its cost-effectiveness and increasing antibiotic resistance may be problematic [6]. These shortcomings, including treatment failure, recurrence and the development of antibiotic resistance, have prompted the search for new antibiotics or other methods for overcoming CDI. The use of lactic acid bacteria (LAB) during antibiotic treatment may transiently change the composition of the gut bacteria, but this reverts to its previous state when LABs are discontinued [7]. Rifaximin and tigecycline (TGE) antibiotics have been recently applied to CDIs, and fecal microbial transplantation is a potential new therapy [8].

Disinfection of contaminated surfaces is a crucial step to prevent the spread within hospitals of pathogens such as *C. difficile.* In addition to traditional manual cleaning, numerous methodologies for surface disinfection have been developed, including disinfectants, automated decontamination devices such as touchless robots that use hydrogen peroxide (HP), ultraviolet (UV) light, self-disinfecting metals (silver and copper), and photocatalysis [9,10,11,12,13,14,15,16]. Although the merits of UV irradiation and HP are their rapidity and very efficient removal of *C. difficile* spores, they have disadvantages including high cost, exposure of staff to these agents, and the suboptimal effectiveness of surface cleansing [12,14,15]. In particular, the wavelength of UV used (UVC, 200–270 nm) seems to be too short to penetrate deeper lesions such as biofilms.

Tetracyclines (TCs) are bacteriostatic antibiotics that have a broad spectrum of efficacy against both Gram-positive and Gram-negative bacterial infections. TCs have a low risk of inducing resistant CDI [17], and prevent bacterial growth by binding to the aminoacyl-tRNA of ribosomes, thereby inhibiting protein synthesis [18]. As a side effect, TC therapy may induce photosensitivity to ultraviolet A (UVA, 315-400 nm) because of its induction of oxygen radical production. Representative toxic effects of TCs include erythrocyte disruption, monocyte injury, virus inactivation, and sunburn. TCs have a strong phototoxic effect against *Escherichia coli*, the mechanism of which is thought to involve superoxide radicals produced by photodynamic therapy (PDT) [19]. This phototoxicity depends on both the light energy and TC concentration. In our previous study [20], we reported for the first time the use of in vitro TC-based PDT against *C. difficile.*

The present study was undertaken in order to determine the capability of TCs to act as an effective photosensitizer (PS) during PDT against *C. difficile*. We compared the PS effect of four commonly used TCs [TC, doxycycline (DXY), minocycline (MIN), and TGE] against *C. difficile* to identify which was the most appropriate PS.

## 2. Results

### 2.1. Absorption Patterns of the Four TCs

**Figure 1:**
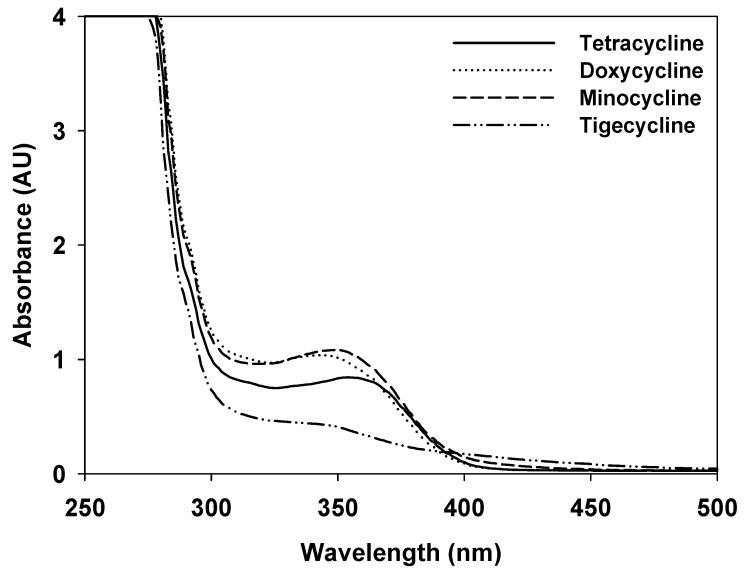
Ultraviolet A absorbance curves of tetracycline (TC), doxycycline (DXY), minocycline (MIN), and tigecycline (TGE).

The absorbance curves of the four TCs were tested and TC, DXY, MIN, and TGE showed maximum absorption at 355, 350, 345, and 340 nm, respectively (Figure 1).

### 2.2. Intrinsic Bactericidal Activity of the Four TCs Against C. difficile in the Absence of Light Irradiation

**Figure 2:**
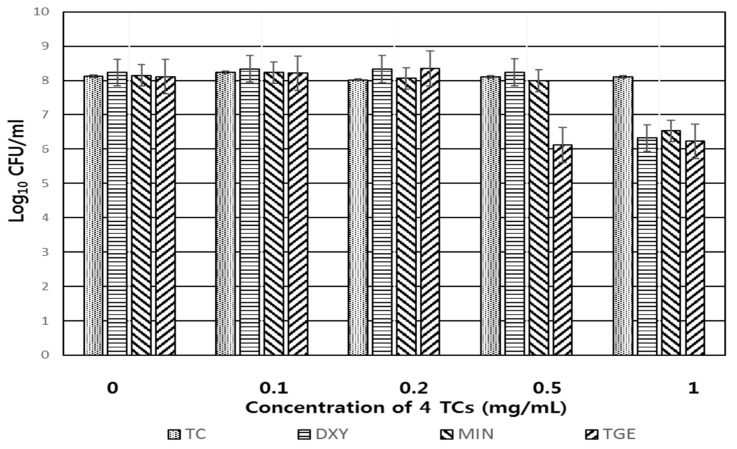
Antimicrobial activity of the four TCs alone against *C. difficile* KCTC5009 after 30 min exposure.

The bactericidal effect of the four TCs against *C. difficile* in the absence of UVA irradiation was evaluated to assess any inherent inhibition by the antibiotic itself. As shown in Figure 2, cells were not influenced until the concentration of the original TC reached 1 mg/mL for at least 30 min. Bacterial cells were not influenced by concentrations of DXY and MIN as high as 0.5 mg/mL, or concentrations of TGE as high as 0.2 mg/mL. The combined antimicrobial activity against *C. difficile* of the four TCs plus chitosan without UVA was subsequently determined at low TC concentrations (0.05 and 0.1 mg/mL).

### 2.3. Comparison of the Effect of the Four TCs on C. difficile in the Presence of Chitosan

The bactericidal effect of PDT increased proportional to the concentration of TCs and the duration of exposure to light (Table 1 and Table 2). In either control conditions or in the presence of chitosan for 30 min, there was little change in the number of live cells. Treatment with UVA, UVA + chitosan, 0.05 mg/mL of three TCs (TC, DXY, and MIN) + UVA, or 0.1 mg/mL of the same three TCs + UVA, resulted in a tenfold reduction in the number of viable cells after 30 min irradiation. The number of live cells was also reduced tenfold by UVA irradiation for 10 min after treatment with 0.05 and 0.1 mg/mL of TGE. Treatment with 0.05 mg/mL of three TCs (TC, DXY, and MIN) + chitosan resulted in a tenfold reduction in the number of viable cells after 20 min irradiation. Treatment with 0.05 mg/mL of TGE + chitosan resulted in a tenfold reduction in the number of viable cells after 10 min irradiation. Treatment with 0.1 mg/mL each of the four TCs + chitosan resulted in a 1,000-fold reduction in the number of viable cells after 5 min irradiation.

### 2.4. Ethidium Bromide Monoazide Quantitative Polymerase Chain Reaction (EMA–qPCR) Analysis for the Evaluation of C. difficile membrane damage

The degree of cellular membrane damage was tested using EMA–qPCR, which can differentiate the DNA from intact and injured bacterial cells. The target gene was the 16S rRNA housekeeping gene. Cellular damage was much higher after treatment with PDT + chitosan than after treatment with PDT alone or under control conditions; the threshold cycle (Ct) values in EMA–qPCR were 14.07 ± 0.22 for the control and 19.45 ± 0.05 for the UVA + chitosan group. In the TCs + UVA groups, the Ct values were 20.46 ± 0.12 to 21.43 ± 0.01. In the TCs + UVA + chitosan groups the Ct values were 24.17 ± 0.08 to 25.54 ± 0.17 (Table 3). However, there was little difference between any of the four TCs (Table 3).

### 2.5. Evaluation of Membrane Integrity

**Figure 3:**
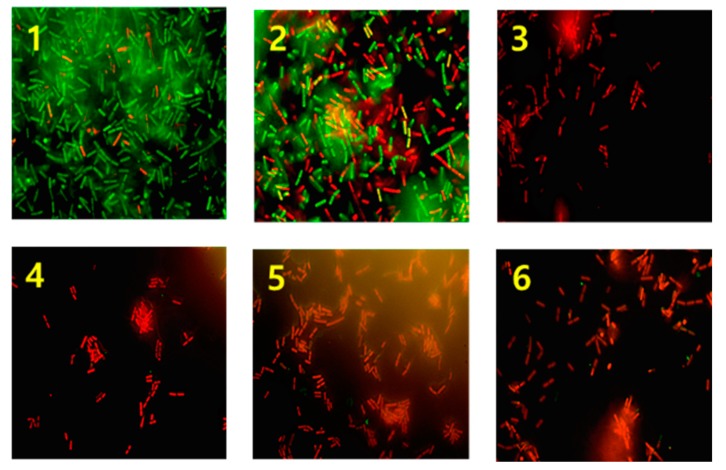
Fluorescence microscopy: the difference in bacterial membrane integrity after photodynamic therapy with UVA plus chitosan. 1. Control, almost green colored live cells stained with SYT09 are visible. 2. Irradiation with UVA only. 3. Irradiation of UVA with 0.2mg/mL of TC + chitosan. 4. Irradiation of UVA with 0.2 mg/mL of DXY + chitosan. 5. Irradiation of UVA with 0.2mg/mL MIN + chitosan. 6. Irradiation of UVA with 0.2 mg/mL of TGE + chitosan. In case of image 2-6, red cells indicate dead bacteria detected by the dye (propodium iodide) penetration via the damaged membrane with PDT for thirty min.

The membrane integrity of *C. difficile* was measured after PDT via fluorescence microscopy by assessing membrane permeability using different-colored fluorescent DNA probes. The control group showed almost green-stained cells, indicating an intact cell membrane. The red cells observed in the control group are considered naturally dead cells. After treatment with UVA for 30 min, cells showed a mixture of green and red fluorescence and the numbers of red cells are increased compared with control group. The same result was also observed in case of chitosan treated group (data was not shown). However, in the groups treated with UVA + TCs + chitosan for 30 min, only red-stained cells were found indicating the presence of only ruptured dead cells (Figure 3). Thus, treatment with TC plus chitosan under UVA irradiation caused severe damage to *C. difficile*.

## 3. Discussion

During recent decades, there has been outstanding progress in the treatment of healthcare-associated microbes, although problems remain to be overcome [12,21,22]. In particular, touchless technologies, such as automated UV-radiation devices and hydrogen peroxide therapy performed in closed rooms, have demonstrated highly effective eradication of pathogens irrespective of the infected location [18,20,22]. Antibiotics have been the most powerful medications for controlling bacterial infections. However, the emergence of bacterial resistance to antibiotics has changed this. To overcome this setback, it has been necessary to initiate exploration for new antibiotics or other methods of control. Photodynamic antibacterial chemotherapy (PACT) is one alternative method to overcome antibiotic-resistant strains of bacteria. Previous studies have reported the use of 5-aminolevulinic acid with gentamicin and Rose Bengal with methicillin to fight resistant bacteria and biofilms [23,24]. Our present study differs in that, unlike in previous studies, TC antibiotics themselves were used as the PS, rather than using antibiotics combined with a separate PS. In the present study, we performed an in vitro study of PDT using TCs plus chitosan to establish a feasible tool to manage surfaces contaminated with planktonic form of *C. difficile* efficiently.

TCs are bacteriostatic antibiotics that are used to treat a variety of bacterial infections. Like vancomycin, TCs have a low risk of inducing resistant CDI [2], because they prevent bacterial growth by inhibiting the binding of aminoacyl-tRNA to the aminoacyl site (A) of the ribosome, thereby inhibiting protein synthesis [18]. TCs are known to have side effects such as phototoxicity under UVA irradiation. The major mechanism inducing TC-related phototoxicity is the generation of oxygen radicals. In our previous study [20], in vitro TC-based PDT combined with chitosan had a marked synergistic bactericidal activity against *C. difficile*. The photosensitivity reactions induced by TCs may vary according to the structure of each drug. Clinical reports have suggested that chlortetracycline has the highest phototoxicity against human lymphocytes [25,26,27], and DXY has greater phototoxicity in healthy human volunteers than other TCs. However, we did not investigate the relationship of the phototoxicity of TCs with their structure. In this study, we compared the photodynamic activity of the four TCs against *C. difficile* as a candidate environmental cleanser for use in healthcare units.

Antibiotic activity against *C. difficile* by the four TCs alone was seen at 2 mg/mL (TC), 0.5 mg/mL (DXY, MIN), and 0.2 mg/mL (TGE), respectively. To elucidate the antimicrobial activity of TCs themselves, we tested PDT of four TCs at low concentrations (0.05 and 0.1 mg/mL).

Treatment with 0.05 mg/mL of the four TCs without the addition of chitosan reduced by tenfold the number of live cells under irradiation of 30 min of UVA. In contrast, the addition of chitosan to TCs resulted in a tenfold reduction in the number of live cells after 20 min. Treatment with 0.1 mg/mL of the four TCs alone decreased the number of live cells after 30 min irradiation by tenfold, but with the addition of chitosan, viable cells were reduced by 1000-fold after only 5 min irradiation. These data confirm that chitosan treatment before TC administration has a synergistic effect on the killing of *C. difficile*, although the bactericidal effects of TC, DXY, and MIN were similar, while TGE was slightly more effective than the other TCs (Table 1 and Table 2). Thus, in the present study, we identified the synergistic effects of chitosan during PDT. We reported previously that chitosan treatment before PDT could enhance its bactericidal effects, which could decrease the required concentration of PS so as to avoid any toxicity [20,28,29]. Chitosan, which can be used in coatings, is biocompatible and has antimicrobial activity. Its antibacterial effects are dependent on factors including its molecular weight, pH, and degree of deacetylation [30]. During the PDT for environmental cleaning, two points may be expected: positively, many other pathogens closely associated with nosocomial infection such as *E. coli* and *S. aureus* could may be killed in parallel with PDT [31,32], whereas negatively, usage of low dose of TCs for PDT would lead to development of TCs-resistant bacteria.

We used EMA–qPCR to measure the degree of bacterial cell damage. EMA is a DNA-binding dye that preferentially binds to double-stranded DNA and is used to discriminate intact DNA from damaged DNA by qPCR. Chitosan treatment allows EMA to enter easily via the bacterial surface to integrate into cellular DNA, which inhibits DNA amplification by PCR [33,34]. Intact cell membranes that are not affected by PDT do not react to the EMA. Therefore, PCR following EMA treatment is able to discriminate damaged from intact cells. The Ct values in EMA–qPCR were 14.07 ± 0.22 for the control and 19.45 ± 0.05 for the UVA + chitosan group. In the TCs + UVA groups, the Ct values were 20.46 ± 0.12 to 21.43 ± 0.01. In the TCs + UVA + chitosan groups, the Ct values were 24.17 ± 0.08 to 25.54 ± 0.17 (Table 3). These results suggest that the enhanced effect induced by chitosan application plus PDT with TCs destroys the cellular structure, which permits EMA to enter the cell freely. However, we found no differences between the four TCs.

Membrane integrity is another parameter affecting bacterial viability. We used a commercially available BacLight Bacterial Viability and Counting Kit (Molecular Probes Inc., Eugene, OR, USA) to assess membrane integrity. The control group showed almost green-fluorescent bacterial cells, indicating intact live cell. Few red cells that observed in control group were considered naturally dead cells. In the UVA-treated group, green cells were mixed with increased numbers of red cells, meaning that this approach is capable of destroying the cell membrane to some extent. In contrast, the groups treated with UVA + four TCs + chitosan showed almost red cells, suggesting the injury or complete disintegration of the cell membrane (Figure 3).

In this study, we did not study the effect of PDT against *C. difficile* spore as the more resistant one than vegetative form. However, because the presence of *C. difficile* spores in the hospital environment is more likely associated with acquisition rather than vegetative cells, it is necessary to investigate in a near future the effect of TCs-based PDT against *C. difficile* spore by the alteration of killing processes such as elevation of light energy and exposure duration. 

In summary, we have shown for the first time the ability of PDT with four TCs combined with chitosan to kill *C. difficile*. The TCs exhibited this bactericidal activity at low concentrations, which could avoid pharmacological side effects. The four TCs studied here did not differ in their photosensitizing and bactericidal activities, but TGE may be preferred as a candidate for PDT. Based on these findings, we propose that TCs might be effective alternative agents for cleaning hospital environments via PDT in the presence of chitosan.

## 4. Materials and Methods

### 4.1. Bacterial Strain and Culture Condition

The type strain of *C. difficile* KCTC 5009 (ATCC 9689, ribotype 001, producing cytotoxin TcdA and TcdB) was obtained from the Korean Collection for Type Cultures (KCTC, Jeongeup, Korea). *C. difficile* was cultivated in strict anaerobic condition at 37°C. The media for *C. difficile* culture was Clostridium difficile agar (KisanBio, Kyunggido, Korea).

### 4.2. Chemicals and Instruments

TC (Cat. No. T7660), DXY (Cat. No. D34477), MIN (Cat. No. M9511), TGE (Cat. No. PZ002142), chitosan with a molecular weight 50,000–190,000 Da (Cat No. 448869), and ethidium bromide monoazide (EMA) were obtained from Sigma (Sigma-Aldrich Co., St. Louis, MO, USA). For the preparation of a stock solution of four TCs, each TC was dissolved in distilled water to 10 mg/mL (1%), filtered with 2 μm filter and refrigerated until use. Chitosan was prepared as described in a previous report [20]. UVA lamp emitting wavelengths in the range 315–400 nm, which is used to treat psoriasis, was used as light sources (UV801KL, Waldman Medical Division, Villingen-Schwenningen, Germany). The irradiation of UVA was performed according to the previous report [20]. The dose of UVA measured using an 843-R Optical Power Meter (Newport Corp., Irvine, CA, USA) was 2.5 mW.

### 4.3. Spectrophotometric Measurement of Absorbance Patterns of the Four TCs

A spectrophotometer (SpectraMax i3x, molecular devices, USA) was used to measure the absorbance curve of each of the four TCs.

### 4.4. Antimicrobial Activity Against C. difficile of the Four TCs Alone

In order to assess the anticlostridial activity of the four TCs during PDT, bacterial cells were exposed to various concentration of four TCs for thirty min. To remove remaining antibiotics bacteria were washed with PBS 3 times and serially diluted bacterial suspension were spotted on the media. After incubation for 48 h, viable cells were counted.

### 4.5. Augmented Photodynamic Anticlostridial Activity of Chitosan and the Four TCs during PDT

This experiment was conducted according to the method described by Tegos et al. [35] and our previous study [20] with slight modifications. In brief, bacterial cells were inoculated onto Clostridial difficle agar and incubated under anaerobic conditions for 48 h. Cell suspensions were prepared in PBS at a concentration of 10^8^ cells/mL. Under UVA irradiation, the cell suspension was exposed to each of the four TCs at concentrations of 0.05 mg/mL or 0.1 mg/mL for 30 min, or to 0.0125% chitosan plus each of the TCs at the same concentrations. These concentrations of TCs were selected to avoid the intrinsic bactericidal activity as determined in our previous study [20]. 

### 4.6. Evaluation of DNA Damage Using EMA-qPCR

To evaluate the degree of cellular damage in the irradiated cells, qPCR was conducted after EMA treatment of cells exposed to PDT with chitosan. This experiment was conducted at high concentration of TCs (0.2 mg/mL) for a definite comparison. After treatment of cells with 0.2 mg/mL of each of the four TCs alone or each of the four TCs + chitosan, they were UVA irradiated for 30 min, then each cell suspension was treated with 100 μg/mL of EMA. The cells were incubated in a dark room for 5 min and subsequently exposed to light from a 650-W halogen lamp 20 cm above the tube for 1 min. The tubes were dipped into ice before exposure to light to diminish any elevation of temperature. The procedure for the preparation of DNA and real-time PCR were performed according to the previous study [20]. This test was executed using housekeeping gene of 16S rRNA.

### 4.7. The Evaluation of Membrane Integrity After PDT With Chitosan

The membrane damage to bacterial cells during PDT was observed with fluorescence microscopy using fluorescent DNA probes according to membrane permeability (BacLight Bacterial Viability and Counting Kit; Molecular Probes Inc., Eugene, OR, USA). Damage to the bacterial cell membrane was judged a sign of cell death. While membrane-permeable DNA probe SYTO9 in viable cells emits green fluorescence (>505 nm), membrane impermeant propidium iodide (PI) in dead cells emits red fluorescence (>633 nm). The experimental procedure to evaluate the effects of chitosan during PDT, was performed according to the previous study [20]. To compare green and red cells, specimen was observed under FITC filter (green, counter stain) preferentially and then changed to Cyc3 filter (red).

### 4.8. Statistics

All values are expressed as the mean ± standard error (S.E.). Statistical differences were evaluated using the student’s t-test. 

## Figures and Tables

**Table 1:** Comparison of augmented photodynamic activity against *C. difficile* of chitosan (Chi) and 0.05 mg/mL of four tetracyclines (TCs).

Time (min)	Log_10_ CFU/mL (mean ± SD, n = 3)											
	Control	Chi ^a^	UVA	UVA + Chi ^a^	Under UVA irradiation							
					TC	TC + Chi ^a^	DXY	DXY + Chi ^a^	MIN	MIN + Chi ^a^	TGE	TGE + Chi ^a^
0	8.13 ± 0.43	8.13 ± 0.22	8.03 ± 0.78	8.03 ± 0.56	8.03 ± 0.22	8.03 ± 0.55	8.03 ± 0.51	8.03 ± 0.55	8.13 ± 0.11	8.02 ± 0.44	8.12 ± 0.55	8.12 ± 0.65
10	8.24 ± 0.56	8.24 ± 0.31	8.13 ± 0.45	8.13 ± 0.77	8.13 ± 0.35	8.13 ± 0.32	8.13 ± 0.23	8.13 ± 0.65	8.23 ± 0.15	8.26 ± 0.58	7.45 ± 0.49	6.99 ± 0.33
20	8.11 ± 0.23	8.05 ± 0.33	8.12 ± 0.49	7.52 ± 0.65	8.11 ± 0.44	7.11 ± 0.22	8.11 ± 0.33	7.03 ± 0.33	8.12 ± 0.54	7.12 ± 0.23	7.55 ± 0.41	6.79 ± 0.23
30	8.12 ± 0.32	8.12 ± 0.35	7.12 ± 0.44	7.12 ± 0.15	7.12 ± 0.72	7.12 ± 0.14	7.12 ± 0.34	7.03 ± 0.21	7.15 ± 0.65	6.99 ± 0.47	6.89 ± 0.44	7.02 ± 0.74

^a^ = 0.0125% chitosan was used.

**Table 2:** Comparison of augmented photodynamic activity against *C. difficile* of chitosan (Chi) and the four TCs (0.1 mg/mL).

Time (min)	Log_10_ CFU/mL (Mean ± SD, n = 3)											
	Control	Chi ^a^	UVA	UVA + Chi ^a^	Under UVA Irradiation							
					TC	TC + Chi ^a^	DXY	DXY + Chi ^a^	MIN	MIN + Chi ^a^	TGE	TGE + Chi ^a^
0	8.13 ± 0.43	8.13 ± 0.22	8.03 ± 0.78	8.03 ± 0.56	8.03 ± 0.14	8.03 ± 0.55	8.03 ± 0.23	8.03 ± 0.33	8.03 ± 0.22	8.03 ± 0.24	8.03 ± 0.44	8.03 ± 0.59
10	8.24 ± 0.56	8.24 ± 0.31	8.13 ± 0.45	8.13 ± 0.77	8.13 ± 0.27	5.23 ± 0.23	8.13 ± 0.25	4.98 ± 0.54	8.13 ± 0.34	5.23 ± 0.17	7.03 ± 0.14	4.89 ± 0.57
20	8.11 ± 0.23	8.05 ± 0.33	8.12 ± 0.49	7.52 ± 0.65	8.11 ± 0.57	5.12 ± 0.51	8.11 ± 0.47	5.05 ± 0.14	8.11 ± 0.41	5.11 ± 0.14	7.05 ± 0.22	4.88 ± 0.11
30	8.12 ± 0.32	8.12 ± 0.35	7.12 ± 0.44	7.12 ± 0.15	7.12 ± 0.35	5.12 ± 0.14	7.12 ± 0.56	5.12 ± 0.25	7.12 ± 0.56	5.03 ± 0.23	7.23 ± 0.16	4.58 ± 0.24

^a^ = 0.0125% chitosan was used.

**Table 3:** Ethidium bromide monoazide quantitative polymerase chain reaction (EMA-qPCR) analysis of photodynamically damaged *C. difficile* KCTC 5009 DNA.

DNA Samples	Ct Values (Mean ± SD, n = 3)
Control ^a^	14.67 ± 0.22
UVA ^b^	15.60 ± 0.00
Chitosan ^c^	17.46 ± 0.12
UVA + chitosan ^d^	19.45 ± 0.05
UVA + TC ^e^	20.46 ± 0.12
UVA + TC + chitosan ^f^	24.17 ± 0.08
UVA + DXY ^e^	21.43 ± 0.01
UVA + DXY + chitosan ^f^	25.28 ± 0.01
UVA + MIN ^e^	20.25 ± 0.03
UVA + MIN + chitosan ^f^	25.31 ± 0.03
UVA + TGE ^e^	21.38 ± 0.10
UVA + TGE + chitosan ^f^	25.54 ± 0.17

^a^ = DNA from *C. difficile* KCTC5009 without TC, Chitosan, and UVA; ^b^ = DNA from *C. difficile* KCTC5009 treated with UVA only; ^c^ = DNA from *C. difficile* KCTC5009 treated with chitosan (0.0125%) only; ^d^ = DNA from *C. difficile* KCTC5009 treated with UVA and chitosan (0.0125%); ^e^ = DNA from *C. difficile* KCTC5009 treated with UVA and each of the four TCs; ^f^ = DNA from *C. difficile* KCTC5009 treated with UVA, each of the four TCs, and chitosan (0.0125%).
